# Notes and News

**Published:** 1887-01-22

**Authors:** 


### NOTES AND NEWS.

COLLECTED AND COMMUNICATED.

LORD VERNON has succeeded Sir Andrew Walker as President of the Children's Hospital at Derby.

THE building fund of the new Swindon Hospital already amounts to over £700, and a special appeal has been made to the local clergy to assist the Committee by the contribution of an offertory-collection. It is anticipated that the hospital will be built and opened by the fall of the year. The plans have been prepared by Mr. W. H. Read.

MR. VERDIN, M.P. for Northwich, has announced his intention of presenting that town with a house, formerly a gentleman's residence, and the gardens belonging to it. The house is to be converted into a children' hospital, and to be called for the future "The Victoria Infirmary," whilst the gardens will supply ample playground for the convalescent patients.

THE Town Councillors of Hereford have been deliberating over no less than eight proposals made for a local memorial of the Jubilee Finally, they decided that the best permanent memorial would be either a new Eye and Ear Infirmary, a Children's Ward at the Infirmary, or a public hall. The ratepayers are going to be polled for a decision.

THE Committee of the Boston Cottage Hospital are very anxious to add a "Jubilee Wing" to their institution, if the funds will admit of it. The patients are so increasing in number that an extension of the hospital will soon become an imperative necessity. When so many other towns in Great Britain intend to celebrate this year of Jubilee by providing for their sick poor, we do not believe the people of Boston will be left behind.

A MOVEMENT is on foot at Yarmouth to erect a Fisherman's and Cottage Hospital at Gorleston. An executive committee, including the vicar of Gorleston, has been appointed to go into the question of site, cost, etc., and to take the necessary steps to bring the matter before the public. Mr. H. Harvey-George is acting as hon. sec. *pro tem.*

THERE is some talk of commemorating the Queen's Jubilee in St. Helen's by the erection of a Free Infirmary. St. Helen's is now a large, populous, and important town, and has quite outgrown the existing hospital provision. It could not, we should think, place on record a better memorial to that queen, of whom Tennyson wrote "She wrought her people lasting good," than by placing itself at least on an equal footing with the other great manufacturing centres of South Lancashire in the matter of hospital accommodation.

ON Wednesday 9th prox., the Lord Chancellor (Lord Halsbury) has promised to take the chair at a festival dinner at the Holborn Restaurant, in aid of a Jubilee Fund for opening the whole of the wards of the new building—known as the Albany Memorial—of the National Hospital for the Paralysed and Epileptic, Queen's Square, Bloomsbury.

OF the almost countless proposals which peeple are making in respect to Her Majesty's Jubilee commemoration, it is exceedingly gratifying to observe how large a share of thought has been directed to the hospitals. The Queen has herself ever taken a deep interest in these noble institutions, and, throughout her long and glorious reign, whenever any sad accident has fallen upon her people, she has never failed to tender her condolence, and to make sympathetic inquiry as to the condition of the sufferers. We believe that no form of memorial would be more pleasing to the Queen than that which handed down her name, to generations yet to come, in associations with the hospitals of our land.

SIR ARTHUR HAZLERIGG presided at the Quarterly Meeting of the Governors of Leicester Infirmary, on the 11th inst., when the Treasurer, Mr. Fielding Johnson, stated that, for the first time for many years, he had the good fortune to report a balance in hand on the infirmary account. This balance amounted to over £400. The Saturday collection among the working-classes in Leicester has been steadily improving, and last year it reached the total of £1,982, an increase of £253 on the collection in 1885. It is satisfactory to note that the working-men of Leicester are still, and even increasingly, alive to the beauties of charity and compassion; so that there is hope, despite all reports to the contrary, that the savagery of Communistic doctrine has not yet gained much hold amongst them.

The nurses at the Worcester Infirmary have been supplied with a piano for their amusement during the winter months. Whilst believing that every reasonable relaxation should be allowed to so meritorious a class, we cannot think that the introduction of musical instruments into a hospital is desirable, unless the nurses' room is so situated as to prevent the sounds from being heard by the patients in the wards. Otherwise, if many people in good health experience unreasonable and excessive annoyance from the sounds produced by these instruments, as they undoubtedly do, then the effect of such sounds upon nervous and irritable patients would in all probability be injurious to an almost incalculable extent.

During the past Christmas, some thousands of mottoes decorating the walls of hospitals have come under our notice. Some of them departed from the customary Christmas greeting. Some of them welcomed the doctors, the matrons or the nurses, but the palm for singularity in the matter of mural decoration we must certainly award to the Mineral Water Hospital at Bath, where in the King's ward, was the quaint inscription: "Long life to our doctors, and may they never have the gout."

The following vacancies are announced this week, the amount of the salary being placed in each case after the name of the institution :—

**Honorary Medical Officers.**

Chelsea Hospital for Women. Assistant Physician Registered.
Great Northern Central Hospital. Surgeon, F.R.C.S.E.

**Resident Medical Officers.**

Bedford General Infirmary. Surgeon, doubly qualified. —£100, with board, etc.
East Suffolk Hospital. House Surgeon, doubly qualified. —£100, with board, etc.
Jaffray Hospital, Birmingham. One, doubly qualified.—£150, with board, etc.
London Temperance Hospital, N.W. Junior House Surgeon. —Board, etc
Retford Dispensary. Surgeon, fully qualified.—£120, without board.
Seaman's Hospital, Greenwich. House Surgeon, registered. —£50, with board.
Worcester Infirmary. House Surgeon, doubly qualified.—£100, with board.

**Trained Nurses.**

Dundee Lunatic Asylum. Head Female Attendant.—£40, with board.
Leeds Fever Hospital. One Charge, £30. Several Assistants, £20 to £25. Uniform.
Monsall Fever Hospital, Manchester.—£30, with uniform.
Maidstone Hospital. Lady. £25, without uniform.
Middlesbro' Infirmary. One.—£25, with uniform.
Ryde Royal Infirmary. Night.—£20, without uniform.

---

An interesting item in the report just issued by the directors of Chalmers' Hospital, Edinburgh, is the record of their thanks to "A grateful patient of Dr. Heron Watson" for the munificent donation of £500. If all hospital patients in easy circumstances were grateful! But alas,

"Oft expectation fails, and most oft there
Where most it promises."

---

Mr. A. W. Jephson writes : "Instead of a silly Church House, could not the Queen's Jubilee be kept in mind by paying off the debts on our hospitals? Some have had to sell their invested capital. To start them all free of debt in 1888, would be the best Jubilee Memorial I can think of." We fear that the most sanguine could scarcely anticipate so much as this. Everyone, however, might this year double his contribution to the Hospital Sunday Fund, as a Jubilee offering to the Hospitals. In this way, Mr. Jephson's proposal would be to some extent realised.

---

The total income of the charities of London, during the past financial year, is set down at £4,680,654. Of this vast sum, £895,000 was subscribed to foreign missions, £559,868 to home missions, £547,483 to hospitals, and £107,897 to dispensaries and convalescent institutions.

---

A sub-committee was recently appointed to consider designs for the proposed new hospital near Johnstone, N.B., which is intended to give accommodation to the five parishes of Johnstone, Kilbarchan, Houston, Erskine, and Inchinnan. Out of seventy-two designs sent in response to advertisements, three were selected by the committee and presented to a special meeting of parish representatives on the 6th inst. for their final decision. This was given, after some discussion, in favour of the plan of Mr. John Woodrow, Bridge of Weir. The new joint hospital is to contain sixteen beds, and a suitable site has been obtained at Muirhead, about a mile from Johnstone, at a yearly rent of £10 per acre. It is believed that the total cost will not exceed two thousand pounds.

---

The late inclement weather has, we fear, done much injury to the cause of the Liverpool hospitals. The 9th inst. was celebrated in that city as "Hospital Sunday," and a more unfortunate fixture could scarcely have been made, for snow fell heavily all over Liverpool and its suburbs during the night and early morning, and, in the more open places, the ground was covered several inches deep. Then a thaw set in, and the streets, more especially in the outlying districts, were soon in such a condition that few people, comparatively speaking, cared to attempt them, and the congregations in the various places of worship were greatly diminished in consequence. Some of the collection returns already to hand show how great a loss has resulted ; thus at Great George Street Chapel, only £134 4*s.* 1*d.* was received, as against £211 16*s.* 6*d.* last year. At St. James's, West Derby, £78 3*s.* 2*d.*, against £120 11*s.* 0*d.* ; at St. Andrew's, Renshaw Street, £136 18*s.* 4*d.*, as compared with £208 8*s.* 0*d.* in 1886, and so on in many cases ; whilst in the few instances where an increase is found, it is very small indeed. We hope that those who were prevented from attending their usual places of worship on the day in question, will show by subscriptions sent to the Treasurer of the Fund that the only cause of their absence was the unwonted severity of the weather.

---

At the Birmingham General Hospital, every year on Christmas Day, the matron and doctors, in conformity with a time-honoured custom, wait at dinner on their subordinates, who of course subsequently "return the compliment." This old custom was duly carried out last Christmas.

---

The New Eye Hospital at Cardiff was formally opened on the 10th inst. by Mr. Alfred Thomas, M.P. for East Glamorganshire, in the unavoidable absence of the Mayor, who had been announced as likely to perform the ceremony. In the course of his remarks Mr. Thomas said that the people of Cardiff and the surrounding district owed a debt of deep gratitude to Dr. Milward, to whom the origin of the new institution was to be attributed. He expressed a hope, and we cordially agree with him, that the inhabitants would exhibit their gratitude by liberal subscriptions to the offspring of Dr. Milward's kindly energy.

---

As a suitable climax to the series of musical entertainments which have been provided for the patients in the Royal Hants County Hospital during the Christmas season, a grand distribution of presents from the Christmas Tree, and a display of *tableaux vivants*, took place on Tuesday evening the 11th inst. Numerous donations to the Christmas Tree have been received from various kind friends of the institution during the past few weeks, and with a result in every way satisfactory. The branches, lighted up by over seventy Chinese lanterns, were weighed down beneath their glittering burden of toys, bon-bons, sweetmeats and the thousand and one trifles usual on such occasions. The *tableaux* were carried out by the sisters and nurses of the hospital, and proved the greatest success. The ward in which the display was held was gaily decorated for the purpose with evergreens, shields, banners, and festoons. Mottoes, too, were to be seen on every side, and the kindly devisers of the entertainment had so far put off their real characters for the time being, as to display in the most prominent position the oft-quoted, but little-heeded injunction, "Throw physic to the dogs."

At the general annual meeting of the Governors of the Lincoln County Hospital, which was held on Thursday the 13th inst., the abstract of accounts for the year ending December 31st last was submitted. Mr. Danby, the secretary, stated that they began the year owing their treasurer £322 4*s.* 11*d.*, and at the close had increased that debt £101 12*s* 0*d.*, thus showing that expenditure had exceeded income by that amount. That the increase was not greater was due to the fact that they had reduced the expenditure, not that the income of the institution had grown larger. The subscriptions continued to diminish year by year, and the past year had proved no exception, although several hundred circulars had been sent out in the neighbourhood. A minor want consisted of the insufficiency of light amusing literature for the use of patients, and of current newspapers a day old.

Attempts to amuse patients at this season of the year are not confined to London or Great Britain, but they extend also to Ireland, so that the United Kingdom presents absolute accord in its sympathy with suffering at this time, despite the preaching of disruption, and the existence of Unionists and Repealers. Thus a concert and magic-lantern entertainment was given lately at the South Infirmary, Cork, through the kindly exertions of Dr. Guisani, for the amusement and instruction of the convalescent patients. Mr. S. H. Newsom, one of the Committee of Governors, brought his own magic-lantern, and himself acted the part of showman, to the infinite delight and amusement of his audience. The musical part of the programme was of high excellence, and gave great enjoyment; the amateur conductor, Mr. J. C. Marks, fulfilling his self-imposed task to the complete satisfaction of all present. There can be no doubt that such entertainments, if not of too protracted a nature, are of great benefit to convalescents, raising their spirits and helping to distract their thoughts from personal anxieties.

A novel idea has been carried out at the bazaar which was opened on the 8th inst., at Hull, in aid of the Children's Hospital in that town. The bazaar is called "The Grand Jubilee Doll Show," and all sorts and conditions of dolls may be seen displayed upon the stalls. There are giants and dwarfs, elderly people and babies, blondes and brunettes, maids and mistresses, mingled pell-mell in the most charming confusion. There are dolls of plump and rounded proportions, who look the perfection of good temper and inanity, and there are thin dolls with faces certainly not distinguished by amiability; whilst musical, talking, crying, and laughing dolls of every variety abound. On one stall there is a group representing Her Majesty surrounded by the ladies of the Court. The Queen, a very handsome blonde, is gorgeously arrayed in pale blue satin and lace, with a tiara of pearls, and is seated in a chair of state. On another stall a doll's wedding is going on; whilst upon a third may be seen the fairy forms of many dancing dolls waltzing together with much apparent delight. All these novelties must be a source of great amusement, and we hope they may produce a large sum for the benefit of the hospital.

We regret to find, from the fifteenth annual report of the Guest Hospital at Dudley, which has just reached us, that during the year ending at Michaelmas 1886, the expenditure exceeded the income by £305 3*s.* 3*d.* The hospital is, therefore, in debt to the extent of £1,138 5*s.* 8*d.*, since there was a previous deficiency of £833 2*s.* 5*d.*, which has not been provided for. However, there is room for hope in the fact that the total income from all sources—£3,023 14*s.* 0*d.*—was £306 9*s.* 8*d.* more than in the preceding year, while the expenditure was less by £105 16*s.* 8*d.*; and we should think that a vigorous effort on the part of the Committee, combined with an earnest appeal to the inhabitants of the neighbourhood, ought to speedily restore the institution to a sound financial condition, or, at all events, very materially reduce the amount of its indebtedness. That it is doing good work, and deserves support, is evidenced by the figures published in Dr. Keep's Medical Officers' Report, which states that 674 patients were under treatment in the wards during the twelve months, of whom 391 were cured, 147 relieved, and 48 died. There were 131 operations of various kinds, with 15 deaths.

Some kind friends of St. Mary's organised a capital little "Christmas" entertainment, on Wednesday week, in the board-room of the Hospital. Among others present were Dr. Broadbent, Mr. Walter Pearce (the Resident Medical Superintendent), Mr. E. P. Cockey (one of the resident medical officers), Mr. Walter Pye, Mr. A. Q. Silcock, Miss Medill (the Matron), and Mr. Pietro Michelli, the Secretary. So full was the room that a large number of visitors had to stand. All the patients attended, who were sufficiently well. The first part of the programme consisted of a well-chosen selection of vocal and instrumental music, in which Dr. Maguire, Mr. Batley, Mrs. Minton Taylor, Mr. Lane, Mr. Liston, and Miss Myers took part. The patients, however, evidenced a decided preference for the comical Christy Minstrel entertainment which followed, the *entrée* of the sable performers brightening up the faces of the patients in a very noticeable manner. The fun of the evening now commenced in good earnest. Mr. A. P. Luff made an excellent "bones", and Mr. R. Handfield Jones, the interlocutor, raised the risible faculties of his audience to the utmost in his humorous ditty, "Mrs. Somebody swallowed a Fly." The comic songs, the funny rebuses, and jokes, made the sick folk forget their ailments. Like good Lord Chancellor More, the patients of St. Mary's enjoyed the "merrie jests," many of which were really capital.

